# Beyond the Genome: Can Epigenetics Forecast Therapeutic Success in Graves’ Disease and Thyroid Eye Disease?

**DOI:** 10.3390/ijms27021116

**Published:** 2026-01-22

**Authors:** Jacopo Manso, Dario Sardone, Vincenzo Marotta, Antonio Stefano Salcuni, Alessandro Brunetti, Claudia Cipri, Silvia Maria Sciannimanico, Lorenzo Piva, Maria Carpentieri, Alberto Falchetti, Fabio Vescini

**Affiliations:** 1 Endocrinology Unit, Oncology Area Department, Azienda Sanitaria Universitaria Friuli Centrale (ASUFC), 33100 Udine, Italy; jacopo.manso@asufc.sanita.fvg.it (J.M.); fabio.vescini@asufc.sanita.fvg.it (F.V.); 2 Department of Medical Surgical and Health Sciences, Cattinara Teaching Hospital, University of Trieste, Strada di Fiume 447, 34149 Trieste, Italy; dario.sardone96@gmail.com; 3 Department of Medicine, Surgery and Dentistry, “Scuola Medica Salernitana”, University of Salerno, 84084 Baronissi, Italy; vmarotta@unisa.it

**Keywords:** epigenetics, Graves’ disease, Graves’ orbitopathy, thyroid eye disease, microRNA, DNA methylation, treatment prediction, glucocorticoids

## Abstract

Graves’ disease (GD) and Thyroid Eye Disease (TED) are autoimmune disorders characterized by significant heterogeneity in treatment response. Up to 50% of GD patients relapse after antithyroid drug (ATD) withdrawal, and a substantial portion of TED patients (20–50%) are resistant to first-line glucocorticoid (GC) therapy. This review evaluates the current evidence on epigenetic modifications as predictive biomarkers to guide personalized treatment. We synthesized recent findings (up to 2025) from PubMed, focusing on DNA methylation and microRNAs (miRNAs). For GD, ATD relapse risk is linked to a persistent “epigenetic memory” in T cells, notably the hypomethylation of Th17-associated genes. Circulating miRNA signatures, including miR-346, miR-23b-5p, and miR-92a-3p, also show promise in predicting remission. For TED, GC sensitivity is strongly correlated with specific circulating miRNAs. High pre-treatment levels of miR-146a predict a positive response (100% positive predictive value), while low levels of miR-224-5p predict non-responsiveness. While DNA methylation is confirmed in TED pathogenesis, its predictive role is unstudied. Major research gaps persist, particularly the near-total absence of data on histone modifications as predictive markers and the lack of epigenetic predictors for new biologics treatments, which currently rely on genetic or pharmacokinetic markers. Epigenetic biomarkers represent a promising frontier for stratifying patients and optimizing therapeutic strategies in Graves’ autoimmunity.

## 1. Introduction

Epigenetics is defined as a stable and heritable change in gene expression without an alteration in the DNA sequence. The main epigenetic mechanisms include DNA methylation, histone modifications and RNA interference through microRNAs (miRNAs) [1]. The process of DNA methylation can lead to the inactivation of specific genes, while certain histone modifications can result in the activation of particular genes. Classically, the addition of a methyl group to cytosine residues, typically at CpG islands within gene promoters results in gene silencing by inhibiting transcription factor binding.

MicroRNAs (miRNAs) are a class of small, non-coding, single-stranded RNAs that play a pivotal role in the regulation of various biological processes by modulating gene expression at the post-transcriptional level. They have emerged as crucial modulators of immunity and cellular processes.

In general, an RNA molecule of the miRNAs class is known to bind to the 3′ untranslated region (3′-UTR) of the target gene mRNA, thereby either suppressing translation or causing the degradation of the mRNA of numerous transcripts through the process of silencing initiated by the miRNA complex, the basis of which is sequence complementarity [2]. However, less frequently, the same molecules can also stimulate gene expression. It is estimated that up to 50% of all coding genes can be regulated by miRNAs, and each individual gene could be influenced by multiple miRNAs [3].

Graves’ disease (GD) is an autoimmune thyroid disorder characterized by the production of anti-thyroid-stimulating hormone (TSH) receptor antibodies (TRAb) and is the most common cause of hyperthyroidism with a global prevalence of approximately 0.5% in men and 3% in women [4,5].

Thyroid Eye Disease (TED), also known as Graves’ orbitopathy, is the main extrathyroidal manifestation of GD, affecting approximately 25–50% of patients [6]. Its pathogenesis involves a complex interplay of genetic, environmental, and endogenous factors leading to the expansion and remodeling of orbital tissues [7,8]. TED significantly impairs the quality of life, often causing disfigurement, functional visual impairment, and psychological distress [9].

Given that only 50% of patients with GD experience sustained remission after 12–18 months of antithyroid drug (ATD) therapy and that about 50% may relapse in the first 6–12 months after treatment is suspended, the need for prognostic factors to predict remission would be very useful in clinical practice to identify in advance which patients should be given medical therapy rather than definitive therapy (surgery vs. radioiodine) [10,11].

Furthermore, in the context of TED, the response rate to treatment with intravenous glucocorticoids (IVGCs) is between 50 and 80%, indicating that between 20 and 50% of patients undergo a first cycle of IV steroids without benefit [6]. A recent retrospective study of 64 patients with moderate-to-severe TED reported a positive response to IVGCs in 75% of patients based on clinical judgment, and in 73% based on the EUGOGO composite index [12]. Notably, non-responders were older and presented with significantly higher baseline CAS scores (*p* = 0.001). Multivariate analysis indicated that both elevated CAS scores and increasing age were associated with a reduced probability of a positive response (*p* < 0.05). Considering the intrinsic limitations and subjectivity of the CAS, the identification of objective molecular markers is imperative for the reliable prediction of glucocorticoid resistance. In this regard, epigenetics holds promise in demonstrating a therapeutic predictive role in both GD and TED.

## 2. Methodology

To evaluate the role of epigenetics in GD and TED, we conducted a comprehensive literature search on PubMed covering the period from January 2015 to February 2025. The search strategy employed the following keywords in various combinations: “Graves’ Disease,” “Thyroid Eye Disease,” “Graves’ Orbitopathy,” “Epigenetics,” “DNA Methylation,” “microRNA,” “miRNA,” “Histone Modification,” and “Treatment Response.” We prioritized prospective clinical studies, randomized controlled trials, and matched case–control studies. Articles were included if they investigated epigenetic markers in human biological samples (serum, plasma, or tissue) and correlated them with therapeutic outcomes (ATD remission/relapse or glucocorticoids response) or disease pathogenesis. Studies on non-thyroidal autoimmune diseases or purely in silico analyses without validation were excluded.

## 3. DNA Methylation and Histones

### 3.1. DNA Methylation and GD Relapse

Although DNA methylation and histone modifications are emerging as possible epigenetic factors in the development of GD, their role in predicting treatment response has, until recently, been largely uninvestigated. However, significant progress has been made in understanding refractory GD. Recent studies suggest that relapse is linked to an “epigenetic memory” in immune cells [13].

Specifically, studies focusing on T-cell profiles have observed significant hypomethylation in the promoter regions of Th17-associated cytokine genes, namely IL-17, IL-21, and IL-22, in GD patients compared to healthy controls [13]. Critically, in patients who experience relapse or have refractory disease, this hypomethylation persists despite ATD treatment. This persistent epigenetic arrangement suggests that conventional therapy, like methimazole, may control hormone production but is insufficient to reset the underlying immune dysregulation, predisposing the patient to relapse once the drug is withdrawn. Certain CpG sites (e.g., chr4_123542549_R) have shown a direct correlation with TRAb and FT4 levels. From a translational perspective, this specific epigenetic signature offers significant incremental value beyond established clinical and biochemical predictors. While TRAb currently represent the gold standard for diagnosis and the primary biochemical predictor of relapse, their prognostic sensitivity is not absolute, particularly in patients with borderline titers at the time of drug withdrawal [11,14]. TRAb levels reflect the active humoral response but may not capture the latent immunological memory that precipitates recurrence. It can be hypothesized that the persistent hypomethylation of Th17-associated loci acts as a stable epigenetic ‘scar,’ retaining the memory of autoimmunity even when serological markers have normalized, as suggested by differential methylation patterns observed in refractory cases [15]. Consequently, determining the methylation status of Th17-related genes could serve as a risk stratification tool for ‘grey area’ patients—those who are clinically euthyroid with negative or low-positive TRAb levels after a course of ATD. In this clinical setting, detecting persistent hypomethylation would indicate a high likelihood of dormant autoimmunity, guiding the clinician toward prolonged medical therapy or definitive treatment (radioiodine or surgery) rather than premature drug withdrawal [16]. Integrating this epigenetic marker with conventional TRAb measurement could therefore refine the decision-making algorithm in GD management, paving the way for a more personalized predictive approach.

### 3.2. DNA Methylation and Histone Modifications in TED

In contrast to GD, the predictive role of these modifications in TED remains unstudied. Research in TED has focused on pathogenesis, demonstrating that orbital fibroblasts from TED patients exhibit differential gene expression and a different degree of DNA methylation compared to controls, as confirmed by whole methylome analysis [17]. These epigenetic changes are thought to be crucial for the persistence of the disease phenotype over time.

However, a critical gap remains: to date, these specific methylation patterns in orbital fibroblasts have not been correlated with the prediction of response to any specific treatment, such as glucocorticoids. Furthermore, the role of histone modifications (e.g., acetylation and deacetylation) is universally cited as a major unexplored area. Multiple recent reviews conclude that the potential of histone modifications as diagnostic or predictive biomarkers in autoimmune thyroid disease “remain to be investigated” [17,18]. Future research should focus on mapping the methylation status of GC-receptor genes (e.g., *NR3C1*) and inflammatory mediators (e.g., *IL-6*, *ICAM-1*) in orbital fibroblasts. Correlating these epigenetic signatures with clinical outcomes could reveal why orbital fibroblasts in some patients maintain a pro-inflammatory phenotype despite high-dose steroid treatment.

## 4. MicroRNAs: Potential Biomarkers and Modulators

**Figure 1:**
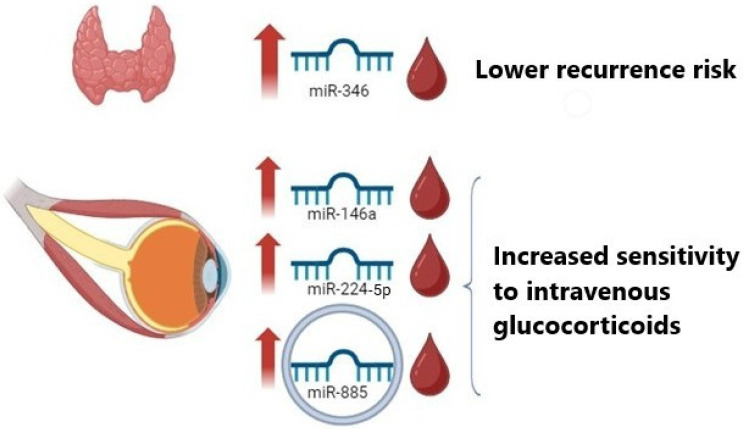
Circulating microRNA signatures predicting therapeutic outcomes in Graves’ Disease and Thyroid Eye Disease. Patients with higher circulating miR-346 at diagnosis of Graves’ Disease showed a lower recurrence rate. Higher levels of circulating miR-146a, miR-224-5p, and exosomal miR-885 were associated with a positive response to glucocorticoids in patients with thyroid eye disease.

In GD and in TED, miRNAs are emerging as potential biomarkers and mediators of pathogenesis. As will be demonstrated subsequently, they can also be utilized to predict the response to glucocorticoids, which are used as a first-line treatment in patients with active TED, or predicting ATD response in GD, Figure 1. MiRNAs have been isolated and quantitatively measured in both tissues and biological fluids, such as serum or blood plasma. This section is divided by disease to analyze the miRNAs involved in treatment prediction.

### 4.1. Predicting ATD Response in GD

A study by Li and colleagues investigated the possible usefulness of miR-346 in predicting relapses in GD patients [19]. In 103 consecutive GD patients treated with methimazole, serum miR-346 levels were measured at diagnosis and at remission. Sixty-seven (65%) of the patients remained in remission and 36 (35%) had a relapse of GD within 1 year of discontinuation of methimazole. Patients with higher levels of circulating miR-346 (≥median value), both at diagnosis and at discontinuation of antithyroid treatment, showed a higher relapse-free survival rate than those with values < median. Consequently, serum levels of miR-346 emerge as a candidate predictive marker of GD relapse, although prospective validation in larger cohorts is required to confirm its clinical utility.

Beyond single markers, miRNA signatures may offer a more robust prediction of remission. A key study by Hiratsuka et al. identified distinct profiles comparing refractory GD patients to those in remission [20]. They found that levels of miR-23b-5p and miR-92a-3p were significantly increased in GD patients who achieved remission. Conversely, levels of let-7g-3p and miR-339-5p were significantly decreased in the remission group, implying that high levels of these two miRNAs are associated with refractory disease. MiR-27a This suggests that, if validated, monitoring a panel of serum miRNAs might offer a dynamic assessment of immunological remission, potentially serving as an adjunctive tool to support the clinical decision of stopping ATD therapy.

It is important to note that these molecular markers must prove superior to, or at least additive to, standard clinical models. A recent 2025 retrospective study developed a predictive model for GD recurrence based only on routine pre-therapeutic clinical data, identifying older age and higher baseline FT4 levels as independent predictors of relapse [21]. Future clinical validation will likely require hybrid models integrating these clinical factors with epigenetic markers (like IL-17 methylation and miRNA signatures) to achieve maximum predictive accuracy.

### 4.2. Predicting Glucocorticoid Response in TED

A significant body of research, summarized in Table 1, has focused on identifying two key sets of miRNAs: those that predict the response to IVGCs (the first-line therapy for active, moderate-to-severe TED) and those involved in TED pathogenesis.

#### 4.2.1. MiRNAs Involved in the Response to Glucocorticoid

MiR-146a has been demonstrated to play a fundamental role in modulating the immune response [25]. In a recent study, we conducted a prospective longitudinal investigation on a cohort of 30 patients with moderate-to-severe active TED undergoing therapy with IVGCs [22]. Prior to the initiation of treatment, circulating microRNA levels were measured in patients’ serum. Notably, elevated pre-treatment serum levels of miR-146a emerged as a predictor of response to glucocorticoid treatment. Furthermore, by means of ROC analysis, a cut-off of 0.56 for miR-146a was identified as capable of predicting responsiveness to IVGCs with a sensitivity of 52.2%, a specificity of 100%, and a positive predictive value of 100%. However, these findings must be interpreted with caution. The study was a pilot investigation with a limited sample size. While the high positive predictive value is promising, suggesting that patients with elevated miR-146a are highly likely to respond, large-scale multicenter validation is required before this biomarker can be implemented in routine clinical practice.

In a further study by Shen et al., circulating miRNAs were analyzed as possible predictors of sensitivity to glucocorticoids in patients with TED (n = 35), finding that treatment-resistant patients had lower pre-treatment serum levels of miR-224 (specifically miR-224-5p) [26]. However, it should be noted that the combination of TSH receptor antibody levels was the only factor capable of achieving a positive predictive value of 91% in terms of predicting treatment response.

Exosomes are vesicles secreted by almost all cell types and in these, microRNAs are protected from degradation. Sun et al. sought to determine the sensitivity to IVGCs in patients with TED. For this purpose, they sequenced miRNAs on plasma exosome samples before starting treatment in patients with TED that were responsive (n = 11) and non-responsive (n = 6) to glucocorticoids [23]. The Authors identified that plasma exosomal miR-885 levels were higher in glucocorticoid-responsive TED patients. Furthermore, through functional experiments in vitro (primary orbital fibroblast cell cultures) and in vivo (mouse models of TED), they demonstrated that (1) the transfer of exosomes from responsive TED patients was able to increase miR-885 levels in orbital fibroblasts with increased expression of glucocorticoid receptors and reduced levels of inflammatory factors (AKT and NFkB), thus increasing sensitivity to glucocorticoids in orbital fibroblasts; (2) in mice with TED responsive to glucocorticoid treatment, the plasma exosomal levels of miR-885 were increased, and at the orbital level the glucocorticoid receptor was more expressed and the expression of AKT and NFkB was reduced. Consequently, plasma exosomal miR-885 represents a potential biomarker for predicting glucocorticoid sensitivity in patients with TED, warranting further investigation to assess its reproducibility in diverse patient populations.

#### 4.2.2. MiRNAs Involved Primarily in TED Pathogenesis

While the following miRNAs have been primarily studied for their role in TED pathogenesis rather than treatment prediction, their modulation of inflammatory and fibrotic pathways suggests they may be indirect indicators of disease state or potential future therapeutic targets.

**MiR-192-5p and miR-484**

In the study by Kim N. and colleagues, the objective was to identify epigenetic biomarkers [27]. The patients with TED were divided into two groups: one with active TED and one with inactive TED. The study involved the analysis of 798 miRNAs, and 10 statistically relevant candidates were subsequently identified. Among these, miR-192-5p, which has been implicated in various biological processes, including cell proliferation, apoptosis, and inflammatory response [28], was found to be significantly reduced in patients with active TED compared to those with inactive TED. However, the ROC curve analysis revealed that the discriminatory power of the investigated miRNAs was enhanced when combined with miR-484 (*p* = 0.121 and *p* = 0.007 vs. *p* = 0.001, respectively) [27]. The modulation of these miRNAs has the potential to influence the processes of inflammation and orbital remodeling, suggesting their possible use as biomarkers of the active phase of the disease.

**MiR-182-5p**

Baiguang Yu and collaborators explored the role of miR-182-5p in CD34+ orbital fibroblasts, a cell population crucial for the pathogenesis of Thyroid Eye Disease (TED), distinct from the lymphocytic infiltrate found in the thyroid gland [29]. A case–control study was conducted in which patients with TED (N = 25) and a group of healthy control patients (N = 24) were enrolled. Initially, the levels of miR-182-5p contained in the orbital fibroblasts of patients with TED were comparable to those of the healthy control group. Subsequently, the patients were further divided into CD34+ and CD34- subgroups, and it was observed that the levels of miR-182-5p were significantly increased in the CD34+ group. The study demonstrated that up-regulation of IL-6 levels in TED patients would favor the expression of miR-182-5p in CD34+ fibroblasts of orbital connective tissue, thus inducing a switch to a profibrotic/proinflammatory phenotype, as previously suggested by some studies in which IL/STAT3 signaling induced the expression of miR-182-5p in Th-17 cells, increasing the production of pathogenic cytokines and promoting cellular autoimmunity [24,30].

**MiR-27a and miR-27b**

Other miRNAs promote the differentiation of orbital fibroblasts into adipocytes, thereby contributing to the development of ocular proptosis in TED patients. Jang SY and colleagues conducted a study to investigate the role of microRNAs-27a and 27b in adipogenesis using an in vitro TED model [31]. These specific miRNAs have been shown to play a role in the metabolism of cholesterol and fatty acids. The expression of miR-27 has been observed to inhibit adipogenesis by blocking the peroxisome proliferator-activated receptor gamma (PPARγ) and CCAAT-enhancer-binding protein alpha (C/EBPα) pathways [32]. The present study explores the impact of miR-27a/b in modulating adipogenic differentiation in patients with TED, comparing them with healthy controls, as well as analyzing the expression levels of key proteins involved in adipogenesis (PPARγ, C/EBPα and C/EBPβ). Ocular fibroblast samples were collected from 13 patients with TED and 8 control subjects, then the cell population was cultured and differentiated into adipocytes. Additionally, the fibroblasts were subjected to transfection with miR-27a/b to ascertain any alterations in the expression levels of the aforementioned genes. Subsequent qRT-PCR analysis revealed a substantial decrease in the levels of miR-27a and miR-27b in the orbital fibroblasts of patients with TED when compared to the healthy controls (*p* < 0.05). Furthermore, the levels of miR-27a/b were found to be elevated in orbital fibroblasts prior to the induction of adipogenic differentiation (day 0) and underwent a progressive decline following the initiation of the adipogenic process (days 4, 7, 10). This observation suggests a regulatory role for miR-27a/b in the suppression of adipogenic differentiation. Following the transfection of orbital fibroblasts with miR-27a/b, the subsequent observations were made: (1) a reduction in the expression of PPARγ, C/EBPα and C/EBPβ (*p* < 0.05); (2) a reduction in lipid accumulation as shown by Oil Red O staining; (3) inhibition of adipogenic differentiation compared to the non-transfected group. These data suggest the role of these miRNAs as inhibitors of the adipogenesis mechanism mediated by PPARγ. Clinically, this implies that therapeutic strategies capable of restoring or mimicking miR-27a/b activity in the orbit could potentially inhibit the pathological adipogenesis that drives proptosis in active TED.

**MiR-101-3p**

The study by Zhu Y and colleagues clarified the implications of microRNA-101-3p as a potential regulator of orbital fibroblast proliferation through the inhibition of the pentraxin-3 protein (PTX3) [33]. PTX3 is an acute phase protein related to innate immunity, mediating the inflammatory response and tissue remodeling [34]. Recent studies attribute a pathogenetic role to the PTX3 in TED, since it would seem to act as a TSH-induced factor within ocular fibroblasts, suggesting a possible implication in TED-related fibrosis [35]. In this case too, orbital adipose tissue samples were taken from patients with moderate-severe TED (CAS 3) and from healthy individuals for the isolation of orbital fibroblasts; subsequently, a miR-101-3p mimic was used to increase its expression in TED-orbital fibroblasts. Finally, the expression of miR-101-3p and PTX3 was evaluated by qRT-PCR and Western blot. The levels of miR-101-3p were significantly reduced in the fibroblasts and orbital tissues of patients with TED compared to healthy control patients (*p* < 0.01), while PTX3 was overexpressed in the fibroblasts of patients with TED (*p* < 0.01).

**MiR-155**

MiR-155 is another miRNA that is involved in the inflammatory response and exhibits context- and cell-type-dependent dynamic expression patterns, resulting in the suppression of diverse gene targets and exerting both anti-inflammatory and pro-inflammatory effects. Li et al. have delineated the anti-inflammatory action of miR-155 in relation to calcium-regulated heat-stable protein 1 (CARHSP1), which plays a role in the formation of foam cells associated with atherosclerosis [36]. Conversely, the expression of miR-155 can be induced by the TLR4/NF-κB pathway, and its upregulation has been demonstrated in both myeloid and activated lymphoid cells [37]. Furthermore, studies have shown that miR-155 can promote inflammation by targeting and suppressing key regulators of the TLR4/NF-κB pathway, such as cytokine signaling regulators (SOCS1) and SH2-containing inositol phosphatase 1 (SHIP1). These are important negative regulatory factors within the TLR4/NF-κB pathway. A recent study by Choi YJ and colleagues proposes a new role for miR-155 as a repressor of ITK (interleukin-2-inducible T-cell kinase), a kinase expressed in T lymphocytes, Natural Killer cells, and mast cells. This function suggests that miR-155 may act as an anti-inflammatory factor in the orbital fibroblasts of patients with TED [38]. Furthermore, the involvement of ITK in the pathogenesis of certain autoimmune diseases had previously been observed [39]. The Authors conducted an experimental study utilizing an in vitro model of TED: orbital adipose tissue samples were obtained from 12 patients with TED and 10 healthy control subjects. The expression levels of miR-155 and inflammatory cytokines (IL-6, ICAM-1, COX-2, ITK, IL-17, TNF-α) were measured using quantitative real-time PCR (qRT-PCR). To assess the effect of cytokines on the expression of miR-155, the cells were treated with interleukin-1 beta (IL-1β) and TNF-α. Finally, the impact of monoclonal ITK and BTK/ITK inhibitor antibodies (ibrutinib) on inflammatory signaling and miR-155 levels was evaluated. The analysis of orbital samples indicated that miR-155 levels were considerably elevated in TED patients compared to controls (*p* = 0.048), suggesting its potential involvement in the pathogenesis of the disease. Furthermore, the exposure of orbital fibroblasts to IL-1β and TNF-α resulted in elevated expression levels of miR-155, a phenomenon observed in patients with TED and in control groups. The effect of IL-1β on miR-155 expression was time-dependent, whereas TNF-α had no significant impact on its expression. The study yielded additional findings which included: firstly, the over-expression of miR-155 using miR-155 mimics led to a significant reduction in the production of IL-6 and ICAM-1 in orbital fibroblasts treated with IL-1β. Secondly, the inhibition of miR-155 resulted in an increase in IL-6 and ICAM-1 expression. Thirdly, the over-expression of miR-155 led to a substantial reduction in ITK mRNA expression and, consequently, ITK protein expression. Collectively, these observations indicate that miR-155 exerts an anti-inflammatory effect on orbital fibroblasts. Conversely, ITK inhibition reduces miR-155 expression, establishing a reciprocal regulatory relationship between the two factors. These findings lay the groundwork for developing novel therapeutic approaches for TED based on miR-155 mimics or ITK inhibitors.

## 5. Discussion and Future Perspectives

Despite the paucity of research in this area, there is an emerging body of literature suggesting that certain miRNAs may have potential as biomarkers for predicting treatment response and risk of recurrence during follow-up in GD and TED. These findings, however, are yet to be validated through large-scale studies. The current literature on this subject is limited to small populations, highlighting the need for further research to confirm the prognostic value of miRNAs in predicting treatment response in GD and TED.

A more profound comprehension of these epigenetic modifications holds the potential to facilitate more precise diagnoses, inform optimal therapeutic strategies, and enhance the accuracy of treatment outcome predictions. However, several critical gaps must be addressed.

The most significant gap in the literature is the near-total absence of data on histone modifications. This scarcity is likely driven by technical limitations: unlike DNA methylation, which is chemically stable, histone modifications are dynamic and labile, requiring immediate processing of fresh tissue. Furthermore, obtaining orbital tissue from active TED patients (who are primarily treated medically) is difficult, limiting sample availability for such complex assays.

Nevertheless, their dynamic nature makes them ideal candidates for reflecting active inflammatory states, yet their potential as diagnostic biomarkers and predictors of treatment outcomes in autoimmune thyroid disease has also not been investigated. This remains the largest unexplored territory.

The therapeutic landscape for GD and TED is rapidly evolving beyond ATDs and GCs toward targeted biologics. However, the search for predictive biomarkers for these new drugs is lagging in the epigenetic sphere. For example, response to Iscalimab (anti-CD40) for GD is predicted by genetic variants of the CD40 gene, not epigenetic markers [40]. Future research must focus on identifying epigenetic predictors (e.g., methylation status of CD40) for these costly and highly specific therapies.

Future studies must transcend single-marker analyses in favor of integrated, high-resolution strategies. A comprehensive multi-omic approach is imperative, synthesizing clinical variables (age, FT4, CAS) with genomic and multi-layered epigenetic profiles, such as DNA methylation, miRNAs, and histone codes. Moreover, the application of high-resolution technologies, including single-cell methylome sequencing, will be essential to deconvolute cell-type-specific epigenetic patterns that are currently obscured in bulk serum samples [13]. While epigenetic in vitro diagnostics are already maturing in oncology and cardiology [41], the field of thyroid autoimmunity must now establish validated biomarkers to fully leverage these technological advancements.

Beyond post-transcriptional regulation, the integration of DNA methylation profiling offers complementary prognostic value. As proposed, detecting persistent hypomethylation in Th17-associated loci could serve as a marker of ‘immunological memory,’ identifying patients at high risk of relapse despite clinical euthyroidism. Future research should aim to combine these dynamic (miRNA) and stable (methylation) markers into integrated predictive algorithms. Such a multi-modal approach would enable clinicians to stratify patients more accurately—identifying those who require aggressive early intervention versus those suitable for conservative management.

It is important to acknowledge the limitations of the current landscape. Most studies reviewed here utilize relatively small sample sizes and lack external validation cohorts. Additionally, the heterogeneity in miRNA normalization strategies across studies poses a challenge for comparing results. Consequently, while the identified biomarkers are promising, they are not yet ready for immediate ‘off-the-shelf’ clinical use.

Finally, the potential reversibility of epigenetic modifications opens the door to novel therapeutic avenues. If specific histone modifications or methylation patterns are confirmed as drivers of orbital remodeling in TED, ‘epidrugs’ targeting these enzymes could theoretically halt or reverse disease progression. Moving forward, the ultimate goal is to incorporate these epigenetic signatures into routine clinical practice, paving the way for a truly personalized precision medicine approach in thyroid autoimmunity.

## 6. Conclusions

The clinical management of GD and TED currently faces a significant “precision gap,” where clinical parameters alone often fail to accurately predict therapeutic outcomes. This review highlights that epigenetics—specifically DNA methylation and circulating miRNAs—stands poised to bridge this gap. We have synthesized evidence suggesting that a persistent “epigenetic memory,” characterized by hypomethylation of Th17-associated genes (e.g., IL-17, IL-21), may underlie the high relapse rates observed in GD patients even after apparent clinical remission. Similarly, in the context of TED, specific circulating signatures such as elevated miR-146a and reduced miR-224-5p levels have emerged as possible, albeit preliminary, predictors of glucocorticoid sensitivity.

Collectively, these findings advocate for a paradigm shift from a “one-size-fits-all” approach to a stratified model where epigenetic profiling informs early decisions between prolonged medical therapy and definitive interventions (e.g., surgery or radioiodine). However, the translation of these biomarkers into routine clinical practice is currently hindered by specific limitations, including small cohort sizes in available studies, the lack of standardized quantification assays, and the largely unexplored landscape of histone modifications (“the histone gap”). Future research efforts must prioritize multi-center prospective studies and integrated multi-omic analyses. Only by rigorously validating these epigenetic fingerprints can we hope to spare patients from ineffective treatments and potential side effects, finally realizing the promise of personalized medicine in autoimmune thyroid disorders.

## Figures and Tables

**Table 1:** Summary of microRNAs and their putative roles in thyroid eye disease. C/EBPα-β: CCAAT-enhancer-binding protein alpha and beta; PPARγ: peroxisome proliferator-activated receptor gamma; PPV: positive predictive value; TED: thyroid eye disease.

MicroRNA	Study	Year	Number of Patients	Putative Role/Key Findings	Classification
miR-146a	Manso et al. [15]	2023	30	High pre-treatment serum levels predict positive response to intravenous glucocorticoids (100% PPV).	Predictive
miR-224-5p	Shen et al. [16]	2015	35	Low pre-treatment serum levels are associated with insensitivity to glucocorticoid therapy.	Predictive
miR-885	Sun et al. [17]	2022	17	Plasma exosomal levels are higher in responders; enhances glucocorticoid sensitivity by targeting the AKT/NF-κB pathway and upregulating the glucocorticoid receptor.	Predictive
miR-192-5p/miR-484	Kim N. et al. [18]	2023	15	Significantly reduced in active TED compared to inactive TED; involved in cell proliferation, apoptosis, and inflammatory response	Pathogenic
miR-182-5p	Yu B. et al. [20]	2025	49	Upregulated in CD34+ orbital fibroblasts; promotes a profibrotic and proinflammatory phenotype, contributing to cellular autoimmunity.	Pathogenic
miR-27a/miR-27b	Jang SY et al. [22]	2019	21	Downregulated in TED orbital fibroblasts; act as inhibitors of adipogenesis by targeting PPARγ and C/EBPα-β pathways.	Pathogenic
miR-101-3p	Zhu Y et al. [23]	2024	20	Downregulated in TED; regulates orbital fibroblast proliferation by targeting Pentraxin-3.	Pathogenic
miR-155	Choi YJ et al. [24]	2022	22	Upregulated in TED; acts as an anti-inflammatory regulator in orbital fibroblasts by repressing interleukin-2-inducible T-cell kinase.	Pathogenic

## Data Availability

No new data were created or analyzed in this study. Data sharing is not applicable to this article.

## Abbreviations

ATD	Antithyroid Drug
CAS	Clinical Activity Score
C/EBP	CCAAT-Enhancer-Binding Protein
CpG	Cytosine-phosphate-Guanine
DNA	Deoxyribonucleic Acid
EUGOGO	European Group on Graves’ Orbitopathy
FT4	Free Thyroxine
GD	Graves’ Disease
ICAM-1	Intercellular Adhesion Molecule 1
IL	Interleukin
ITK	Interleukin-2-Inducible T-cell Kinase
IVGCs	Intravenous Glucocorticoids
miRNA	microRNA
NF-κB	Nuclear Factor kappa-light-chain-enhancer of activated B cells
PPARγ	Peroxisome Proliferator-Activated Receptor gamma
PPV	Positive Predictive Value
PTX3	Pentraxin-3
TED	Thyroid Eye Disease
Th17	T helper 17 cells
TRAb	TSH Receptor Antibodies
TSH	Thyroid Stimulating Hormone
UTR	Untranslated Region
